# DomSign: a top-down annotation pipeline to enlarge enzyme space in the protein universe

**DOI:** 10.1186/s12859-015-0499-y

**Published:** 2015-03-21

**Authors:** Tianmin Wang, Hiroshi Mori, Chong Zhang, Ken Kurokawa, Xin-Hui Xing, Takuji Yamada

**Affiliations:** 1 Department of Biological Information, Graduate School of Bioscience and Biotechnology, Tokyo Institute of Technology, 2-12-1 M6-3, Ookayama, Meguro-ku, Tokyo 152-8550 Japan; 2 Earth-Life Science Institute, Tokyo Institute of Technology, 2-12-1-E3-10 Ookayama, Meguro-ku, Tokyo 152-8550 Japan; 3 Department of Chemical Engineering, Tsinghua University, Beijing, 100084 China

**Keywords:** Enzyme mining, Protein functional annotation, Machine learning, Top-down algorithm

## Abstract

**Background:**

Computational predictions of catalytic function are vital for in-depth understanding of enzymes. Because several novel approaches performing better than the common BLAST tool are rarely applied in research, we hypothesized that there is a large gap between the number of known annotated enzymes and the actual number in the protein universe, which significantly limits our ability to extract additional biologically relevant functional information from the available sequencing data. To reliably expand the enzyme space, we developed DomSign, a highly accurate domain signature–based enzyme functional prediction tool to assign Enzyme Commission (EC) digits.

**Results:**

DomSign is a top-down prediction engine that yields results comparable, or superior, to those from many benchmark EC number prediction tools, including BLASTP, when a homolog with an identity >30% is not available in the database. Performance tests showed that DomSign is a highly reliable enzyme EC number annotation tool. After multiple tests, the accuracy is thought to be greater than 90%. Thus, DomSign can be applied to large-scale datasets, with the goal of expanding the enzyme space with high fidelity. Using DomSign, we successfully increased the percentage of EC-tagged enzymes from 12% to 30% in UniProt-TrEMBL. In the Kyoto Encyclopedia of Genes and Genomes bacterial database, the percentage of EC-tagged enzymes for each bacterial genome could be increased from 26.0% to 33.2% on average. Metagenomic mining was also efficient, as exemplified by the application of DomSign to the Human Microbiome Project dataset, recovering nearly one million new EC-labeled enzymes.

**Conclusions:**

Our results offer preliminarily confirmation of the existence of the hypothesized huge number of “hidden enzymes” in the protein universe, the identification of which could substantially further our understanding of the metabolisms of diverse organisms and also facilitate bioengineering by providing a richer enzyme resource. Furthermore, our results highlight the necessity of using more advanced computational tools than BLAST in protein database annotations to extract additional biologically relevant functional information from the available biological sequences.

**Electronic supplementary material:**

The online version of this article (doi:10.1186/s12859-015-0499-y) contains supplementary material, which is available to authorized users.

## Background

Of the known biological sequences in the post-genomic era, the vast majority have not yet been, and cannot be, characterized by experimentation or manual annotation [1]. For example, Swiss-Prot, a protein database with a manually curated functional annotation, has only 547,085 entries as of December 2014, whereas a comprehensive protein database such as UniProt-TrEMBL, which contains a high-quality computationally analyzed functional annotations and covers most of the known protein sequences, contains tens of millions of members. Therefore, automated annotation is necessary to assign functions to uncharacterized sequences. Enzymes are of special importance owing to their central roles in metabolism and their potential uses in biotechnology [2]. Hence, a greater ability to predict enzyme functions will not only give biologists deeper insight into metabolism in general but also increase the toolkits for bioengineers.

Many novel bioinformatics tools with different bases, such as protein structure [3], functional clustering [4], evolutionary relationships [5] and biological systems networks [6], have been developed for enzyme or protein functional annotations. Many of them perform better [7,8] than conventional approaches like BLAST, which is based on pairwise comparisons of gene sequence similarities to assign functions to new genes [9]. However, BLAST is currently the main approach used in functional annotations [10], whereas many recently developed tools are rarely applied in research projects [7]. Additionally, BLAST-based functional annotations perform poorly when only distantly related homologs with similarities of <30% can be found [11,12]. Furthermore, many proteins recently discovered using metagenomics approaches do not have homologs with high enough amino acid sequence identity levels for reliable functional annotation. For example, in a benchmark study, which used a metagenomics approach focusing on cow rumen–derived biomass-degrading enzymes, it was found that in terms of amino acid sequence identity, only 12% of the 27,755 carbohydrate-activated genes assembled had >75% identity to genes deposited in NCBI-nr, whereas 43% of the genes had <50% identity to any known protein in NCBI-nr, NCBI-env and CAZy [13]. Thus, if novel and combinatorial approaches are used, to what extent, with acceptable precision, can we improve the coverage of the protein annotation? For enzymes, there is a well-established system, the Enzyme Commission (EC) number [14], which describes catalytic functions hierarchically using four digits. As far as we know, although many EC number prediction tools are available, most are limited to performance tests within small datasets and none of them has been used to systematically address the comprehensiveness of enzyme functional annotation in public protein database. Thus, a more specific question, “To what extent we can improve, with an acceptable precision, the coverage of enzyme annotations using EC numbers?” is worth addressing by illustrating the power of approaches whose utility goes beyond BLAST. The insight we obtain can be also generalized to protein annotations for other functional attributes.

Thus, novel approaches with high coverage rates that maintain an acceptable precision are of special interest. Hierarchical or top-down algorithms with a layer-by-layer logic satisfy these requirements [15,16]. Such approaches assign functions only at a level that can be inferred with high confidence. Hence, in many cases, general rather than specific functions (for example, the top level of EC numbers) are assigned to avoid the overprediction of protein functions, such as annotation below the trusted cutoff or inference only from a superfamily, a main problem of current database annotations [17]. Furthermore, this approach is suitable for widely accepted protein function definition systems, such as EC or Gene Ontology (GO), both of which are widely applied metrics systems to consistently describe the functions of gene products [18], owing to their hierarchical structure.

Domains are conserved parts of a given protein’s amino acid sequence and structure that can evolve, function and exist independently of the rest of the amino acid chain. Thus, it has been hypothesized that machine learning with domains as input labels might serve as a powerful approach to predict protein functions [19]. For example, the dcGO database, based on associating SCOP domains or domain combinations with GO terms of protein products, infers the domain or domain combinations responsible for particular GO terms [20]. A domain architecture–based approach might thus be a powerful tool for predicting enzymatic functions. Here, we report on “DomSign”, a top-down enzyme function (EC number) annotation pipeline based on domain signature–derived machine learning. We must emphasize, based on the belief that any reliable protein function prediction tools should depend on multiplicity [21], that our purpose here is not just to present a simple function prediction tool but rather to address the issue of to what extent can the coverage of enzyme annotations by EC numbers be improved, with acceptable accuracy, by methods beyond simple BLAST.

To test the reliability of DomSign, many benchmark enzyme annotation methods were compared with. The performance of DomSign was comparable, or superior, to all of them after exhaustive testing against reliable datasets, such as Swiss-Prot enzymes, suggesting that DomSign is a highly reliable enzyme annotation tool that can identify more enzymes in the protein universe. Furthermore, to expand the number of enzymes retrieved from large datasets, we compared our results with those proteins already assigned EC numbers in the original dataset. More ‘hidden enzymes’ were predicted by DomSign. Thus, DomSign, with >90% accuracy suggested by the tests, can be used to predict a large number of enzymes by assigning EC numbers to proteins in both the UniProt-TrEMBL [22] and Kyoto Encyclopedia of Genes and Genomes (KEGG) [23] bacterial subsection, which, respectively, represent the most complete protein database and best metabolic pathway information collection. DomSign also can be applied to metagenomic samples as exemplified by the Human Microbiome Project (HMP) dataset [24], a comprehensive and well-analyzed metagenomic gene dataset focused on parsing the interactions between commensal microorganisms of humans (human microbiome) and human health. In this case, DomSign not only significantly increased the number of EC-labeled enzymes but also helped to clarify the metabolic capacity of the sample by recovering new EC numbers beyond the official annotation. These results highlight the necessity to develop enzyme EC number prediction projects or, more generally, protein annotation projects with novel approaches akin to DomSign to extract more biological information from the available sequencing data.

## Methods

### Definition of a domain signature

Pfam is a protein domain collection with ~80% coverage of the current protein universe [25], and its Pfam-A subsection is highly reliable owing to its manually curated seed alignment. For our purpose, a string of non-duplicated Pfam-A domains belonging to a protein was defined as its domain signature (DS) and used to predict function(s). Although some research has suggested a potential advantage of involving domain recurrence and order in protein GO assignments [26], our results showed that this simpler DS definition provided a higher coverage for proteins identified in metagenomics studies. When utilizing Swiss-Prot protein DSs to retrieve HMP phase I non-redundant proteins, the coverage was 74.7% when considering domain recurrence and order versus 77.1% with more simple definition. Unlike the GO term assignment used previously [26], recurrence did not lead to a significant difference in coverage as indicated by reconstructing the EC number machine-learning prediction model (Additional file 1) used in this work, whose method is presented in the following part. Thus, because the main aim of our study was to improve enzyme annotation coverage, our simpler DS definition was applied.

### Preparation of the dataset

Swiss-Prot and TrEMBL datasets were downloaded on November 2, 2013, from the Pfam ftp site (version 27.0) from which Pfam-A domains were extracted. Pfam-A Hidden Markov Model (PfamA.hmm) for hmmsearch (version 3.1b1) [27] was accessed from the same site. The HMP phase I non-redundant protein dataset (95% identity cut-off, 15,006,602 entries from 690 samples) [24] was collected from the HMP data processing center (http://www.hmpdacc.org/). A benchmark dataset for unbiased tests was collected from [15] (Supplementary Data 2). The files (gene IDs and sequences in the fasta format) from KEGG were downloaded on March 6, 2014. The EC2GO mapping file [28] was downloaded on June 20, 2014 from the GO homepage. All of these files were further processed as stated below.

#### “Sprot enzyme” dataset

The Swiss-Prot dataset is a protein collection with an exhaustive manually curated—and thus reliable—functional annotation. In this context, it was a good choice working as the training set for comparing prediction model performance by cross-validation. The subset of enzymes in Swiss-Prot with both single EC numbers and Pfam-A domains was termed “sprot enzyme”, encompassing 228,710 entries and 4,216 distinct DSs. This set was used to construct the “Specific enzyme domain signature” dataset as described below and also as a training dataset to build the prediction model for enzyme mining in several general protein databases (TrEMBL, KEGG and HMP).

#### “Sprot protein” dataset

Another subset of Swiss-Prot, which contains all of the Pfam-A proteins with single or no EC numbers, was named “sprot protein”, encompassing 46.8% enzymes (with single EC numbers) and 53.2% non-enzymes (without EC numbers), which covers 99.0% of the Swiss-Prot proteins with Pfam-A domains. This dataset was used for model parameter optimization and performance comparisons against BLAST and FS models (see descriptions below in Methods about FS model) [19].

#### “Specific enzyme domain signature” dataset

To identify enzymes from the protein pool, we further constructed a “Specific enzyme domain signature” dataset. The fundamental idea was to remove non-enzyme-derived DSs from the 4,216 distinct DSs belonging to “sprot enzyme”. Because EC numbers do not cover all enzymes, however, a more reliable non-enzymatic dataset beyond simple proteins without EC numbers needed to be constructed. Briefly, for the proteins without EC numbers in Swiss-Prot, their annotation raw files (‘KW’, ‘DR’ and ‘DE’ lines) were filtered using a catalytic or functional uncertainty–inferring term (‘iron sulfur’, ‘uncharacterized’, ‘biosynthesis’, ‘ferredoxin’, ‘ase’, ‘enzyme’, ‘hypothetic’, ‘putative’ and ‘predicted’) to reliably extract non-enzymes. By this means, we collected 2,901 unique DSs from 157,240 non-enzymes carrying Pfam-A domains. After removing these DSs from the “sprot enzyme” DS set, 3,949 specific enzyme DSs were acquired, covering 95.4% of “sprot enzyme”. This dataset was used for selecting enzyme candidates from a protein pool using the benchmark comparison method and enzyme mining process.

#### “SVMHL unbiased” dataset

To compare the performance of our approach with the SVMHL pipeline (see descriptions below in Methods about SVMHL model) [29], the aforementioned unbiased dataset was further processed to remove, as described in their paper, enzyme sub-subfamilies with fewer than 50 members.

#### “TrEMBL enzyme” and “HMP enzyme” datasets

The TrEMBL raw dataset was filtered to extract enzymes with single EC numbers and Pfam-A domains, producing “TrEMBL enzyme”. Likewise, “HMP enzyme” was constructed from the HMP non-redundant protein set. Pfam-A domains were retrieved by an hmmsearch against PfamA.hmm using the cut_tc cutoff with all other parameters set as default. These two datasets were used as the gold standards to test the reliability of the DomSign-based enzyme EC number annotation prior to the actual enzyme mining of TrEMBL and HMP original datasets. The statistics and usage of the datasets constructed in this work are presented in Additional file 2.

### Prediction model description

Our prediction model consists of two separate steps: enzyme differentiation from the protein pool and EC number annotation based on machine learning. In the first step, proteins in query datasets are recognized as potential enzyme candidates if their DSs are among the aforementioned “Specific enzyme domain signature” set. In the second step, a top-down machine-learning model is developed to predict EC numbers.

**Figure 1:**
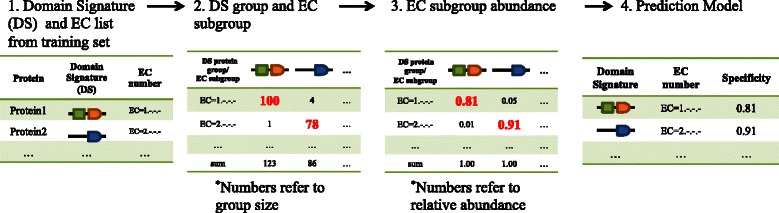
**Construction of the machine-learning model to predict EC numbers.** (1) Test dataset: DSs and EC numbers for every enzyme were extracted from original datasets, such as Swiss-Prot. (2) These proteins were categorized into groups based on common DSs. Subsequently, the groups were divided into subgroups based on the corresponding EC numbers. Thus, the numbers in each cell represent the number of proteins in each subgroup, and the total member number for each group is summarized in the last row. The numbers of dominant subgroups within one group are colored red. (3) The abundance of each subgroup within its parent group (the same DS) was calculated and represented. The abundance of dominant subgroups for each group (the same DS) is colored red. (4) Prediction model: Every DS was associated with the relevant dominant EC number within its protein group (carrying this DS). The abundance of dominant EC subgroups was extracted and set as the “specificity” for this EC-DS pair.

First, we converted the training dataset into a list in which every protein had one DS and one EC number (Figure 1(1)). Subsequently, the proteins were categorized based on their DSs. Thus, we constructed a series of protein groups in which all members contained the same DS. Here, we define the number of member proteins in one group as *N*_*DSi*_. Then, the member proteins in one group were further divided into subgroups based on their EC numbers, leading to a protein subgroup with the same EC (*N*_*DSi* − *ECj*_ and *N*_*DSi*_ = ∑_*j*_ *N*_*DSi* − *ECj*_) (Figure 1(2)). Further, the abundance of every subgroup among one protein group was calculated (*A*_*DSi-ECj*_ *= N*_*DSi-ECj*_ */*_*NDSi*_) (Figure 1(3)). In each group, there exists at least one dominant subgroup with the highest abundance. The EC number for this subgroup is then associated with the relevant DS, whereas the abundance of this subgroup is defined as the “specificity” for this DS-EC pair, which acts as the fundamental parameter in the machine-learning model (Figure 1(4)). We constructed four prediction models to assign four levels for one complete EC hierarchy. For each model, at the first step (Figure 1(1)) one fraction of the EC number was extracted—for instance, for the model focusing on the second EC hierarchy, EC = x.x.-.- is extracted. All further steps were the same during the construction of these four models. Thus, this machine-learning approach makes it possible to annotate the EC hierarchy from general to specific where the “specificity” of DS-EC pairs can be used to balance the tradeoff between recall and precision, depending on the particular purpose.

### From model to prediction engine

**Figure 2:**
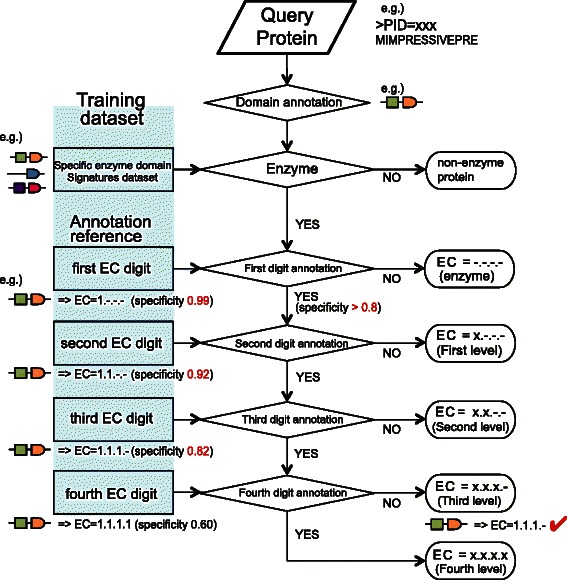
**Schematic representation of the DomSign pipeline.** The pipeline is divided into two parts—enzyme candidate selection and EC number annotation. In the first step, specific enzyme DSs are utilized, and all proteins with DSs within this dataset are selected as potential enzyme candidates. Simultaneously, four annotation references for the EC digits at four levels are constructed as described in Figure 1. At every level, if the “specificity” of the corresponding DS-EC pair in the annotation reference is less than the user-defined threshold, the pipeline is shut down and the previously annotated EC digits form the output. If not, the pipeline continues until the fourth EC digit has been annotated. An example of the DomSign procedure to annotate a protein is shown here. Because the specificity threshold is above the specificity of the DS-EC pair at the last level, only the first three DS-EC digits are predicted, leading to final result: EC = 1.1.1.-.

First, the training dataset was used to construct four prediction models for each EC hierarchy level, and the DSs of query proteins were calculated by hmmsearch with a cut_tc cutoff and all other parameters set as default. Then, the specific enzyme DS dataset was used to select potential enzyme candidates from query proteins. Then, four constructed prediction models were used one by one to annotate EC digits, assigning the EC number that corresponds to the query DS. In this process, a specificity threshold is applied to balance precision and recall. Specifically, when the “specificity” of the DS-EC pair is less than the specificity threshold, the procedure is shut down and only the EC digits annotated previously form the output (Figure 2). In this way, the precision can be increased by making the specificity threshold stricter with a loss of recall, or vice versa. Additionally, although it is not statistically rigorous, the specificity for one particular DS-EC pair can be used as the confidence score to infer the reliability of each prediction by DomSign. For example, if DomSign assigns one enzyme with EC number 1.1.1.- and the specificity values for the DS-EC pair of the first three hierarchical levels are 0.9, 0.88 and 0.85, we can simply set these three parameters as the confidence score for the reliability of prediction for the first three EC digits, respectively. The script package for this tool is provided as Additional file 3.

### Performance evaluation statistics

**Figure 3:**
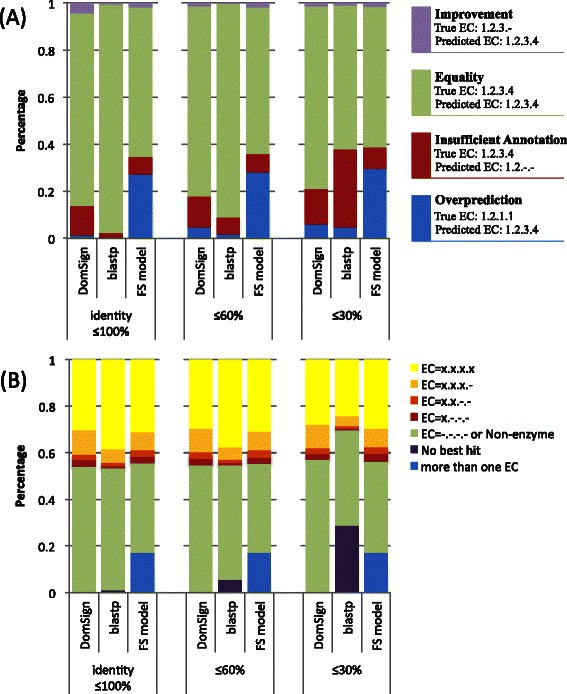
**DomSign performance comparison with BLAST and FS models by 1,000-fold cross-validation of “sprot protein”.** Three levels of 1,000-fold cross-validations were conducted for each method. Homologs of a query above a given threshold (“identity ≤ 100%”, “identity ≤ 60%” and “identity ≤ 30%” described in Methods) were removed from the reference dataset and, for each reference dataset, only sequences below the given threshold were kept. In this test, an 80% specificity threshold, 10^−3^ E-value and default parameters were applied to the DomSign, BLASTP and FS models. The relative standard errors were not significant (<1%) and therefore are not illustrated here. **(A)** Results for the evaluation of the three methods. As shown on the right, four attributes are defined to evaluate the annotation results in contrast to the “true EC number” (see Methods for details). **(B)** The EC hierarchy level distribution in the annotation results of the three methods. Seven attributes are defined here to describe the annotation results. Among them, “No best hit” is specific to BLASTP. “More than one EC” is specific to the FS model because this dataset encompasses only enzymes with single EC numbers or non-enzymes, and this attribute is regarded as “OP” in Panel A. We integrated the annotation result “Non-enzyme” and “EC = −.-.-.-”, as shown in Figure 2 into one unified group, “Non-enzyme”, in the result’s illustration because the latter has no EC number assigned and also only occupies a small fraction of the annotation results (the ratio of the “EC = −.-.-.-” subclass is only 1.4% in the “identity ≥ 100%” group for DomSign) of the annotation results.

Owing to the top-down nature of our approach, we designed a new result evaluation system to use instead of the widely used recall-precision curve [19] that differentiates the annotation results at different levels, resulting in better resolution. Briefly, the predicted EC number (PE) is compared with the true EC number (TE), and the result is classified into the following groups (Figure 3A, right): E—Equality, PE is the same as TE (“PE: EC = 1.2.3.4” vs. “TE: EC = 1.2.3.4”); OP—Overprediction, there is at least one incorrectly assigned EC digit in PE compared with TE (“PE: EC = 1.2.1.1” vs. “TE: EC = 1.2.3.4”); IA—Insufficient Annotation, PE is correct but not complete compared with TE (“PE: EC = 1.2.-.-” vs. “TE: EC = 1.2.3.4”); and IM—Improvement, TE is the parent family of PE (“PE: EC = 1.2.3.4” vs. “TE: EC = 1.2.3.-”). When TE is “Non-enzyme”, if the PE equals “Non-enzyme”, then the comparison result is set as “Equality”. Otherwise, the result is “Overprediction”. Additionally, if PE is “Non-enzyme” and TE is not, then the comparison result is set as IA. What needs to be specifically mentioned here is IA. Although this result means incomplete annotation, it is correct and does not cause any increase in the error rate. Thus, IA provides better annotation coverage and simultaneously maintains high precision. The evaluation metrics defined here differ from traditional ones [19]. However, compared with previous precision-recall curves that equally consider different EC hierarchy levels, this system covers all the possible situations and also gives an intuitive view of the performance at different annotation levels with higher resolution, which is especially suitable for evaluating annotation results using metrics of a hierarchical structure.

### Performance test

Four benchmark methods, BLASTP (2.2.28+), FS [19], SVMHL [29] and EnzML [30], were selected to test the performance of DomSign.

### Comparison with BLASTP and FS by cross-validation

For the FS model, the script package from Forslund K. *et al.* [19] was run on our system to calculate the GO terms derived from the DS defined in their work. Subsequently, we used the EC2GO mapping file to convert the FS model’s predicted GO terms to EC numbers. If multiple EC numbers existed for one particular GO term, we assigned that protein all of the relevant associated EC numbers. The three pipelines were tested by 1,000-fold cross-validations of the “sprot protein” dataset. Because the dataset has only enzymes with single EC numbers or non-enzymes, if the FS model predicted more than one EC number for a query then the result was “OP”. Furthermore, to simulate the situation in which no sequences in the database have a high similarity to the query protein, two additional rounds of cross-validations against “sprot protein” were executed. Briefly, sequences in the training set having specificities above threshold I (60% identity, 80% query coverage) and II (30% identity, 80% query coverage) with any query sequence, respectively, were removed by BLASTP. In this way, any sequence in the training set is no more similar to any query sequence than the defined threshold. These two rounds of cross-validation, together with the common cross-validation, were termed “identity ≤ 100%”, “identity ≤ 60%” and “identity ≤ 30%”. For BLASTP, a 10^−3^ E-value and default parameters were applied. For the FS model, parameters were set as default for the processing.

### Comparison with the SVMHL model by cross-validation

Because the source code of SVMHL is not available, we compared DomSign with SVMHL by the same test as stated in [29], and the raw data were used for performance comparisons. Briefly, a 10-fold cross-validation was conducted using DomSign on the “SVMHL unbiased dataset”, and prediction accuracy [29] was used to evaluate the results. In this case, accuracy is defined as the percentage of completely correct annotations. Here, one predicted EC number at one specific hierarchy level (an EC number consisting of three digits when the EC hierarchy level is three) is set as ‘correct’ when its component digits are all correct. Because SVMHL does not have an enzyme and non-enzyme differentiation step, we included only the predicted “enzyme” by DomSign in the results comparison, which covered 85.2% ± 0.4% of the query proteins on average during the cross-validation.

### Comparison with EnzML

Like the SVMHL model, owing to the inability to run EnzML on our system, we also compared the performance between EnzML and DomSign by the same test stated in [30], and the data published in that paper were used as the benchmark. The “Swiss-Prot&KEGG” set and the less redundant “UniRef50 Swiss-Prot&KEGG” set were constructed according to the description in the EnzML paper [30], and a 10-fold cross-validation was conducted. The example-based precision and recall rate were applied to the performance evaluation. Briefly, these two metrics consider how many correct EC predictions are assigned to each individual protein example on average [31]. For example, for each protein, true (TE) and predicted (PE) EC number sets at every hierarchical level (EC = 1.1.-.- is decomposed to EC = 1, EC = 1.1, EC = 1.1.- and EC = 1.1.-.-) are extracted and compared with each other. The example-based precision and recall rate can be defined by the two equations shown below:

$$ Precision=\frac{1}{m}{\displaystyle \sum_{i=1}^m}\frac{\left|T{E}_i{\displaystyle \cap }P{E}_i\right|}{P{E}_i} $$

$$ Recall=\frac{1}{m}{\displaystyle {\sum}_{i=1}^m\frac{\left|T{E}_i\cap P{E}_i\right|}{T{E}_i},} $$

Here ‘m’ refers to total number of proteins, and TE_i_ and PE_i_ refer to the sets of annotated EC numbers at four hierarchical levels or ‘Non-enzymes’ for each protein.

### Enzyme predictions from large-scale datasets

“Sprot enzyme” was used as the test dataset, and “Specific enzyme domain signature” was used to select enzyme candidates. “TrEMBL enzyme” and “HMP enzyme”, combined with their original annotations, were used to evaluate the reliability of DomSign for expanding enzyme space. All TrEMBL and HMP proteins were then annotated by DomSign to test the extent of the enzyme expansion. Further, to show the significance of enzyme expansion in KEGG, among the predicted novel enzymes of TrEMBL, novel enzymes for 2,584 bacterial genomes in KEGG were extracted. Owing to the subtle differences between KEGG and TrEMBL annotations, a few novel enzymes in TrEMBL have EC numbers in KEGG. These were removed to retrieve the exact number of novel enzymes from KEGG, and the relevant statistics were calculated.

## Results

### Optimization of the DomSign specificity threshold

We tested the reliability of DomSign as an EC number prediction tool. Because we designed a parameter “specificity threshold” (Methods) in DomSign to balance the tradeoff between precision and recall (Figure 2), three rounds of 1,000-fold cross-validations (“identity ≤ 100%”, “identity ≤ 60%” and “identity ≤ 30%” cutoffs as described in Methods) were performed on the “sprot protein” dataset using DomSign with 99%, 90%, 80% and 70% specificity thresholds to optimize this parameter (Additional file 4). Among the 99%, 90% and 80% specificity thresholds, the 80% had the best coverage (IA, E and IM) and a slightly increased error rate (OP). However, further reduction of the specificity threshold to 70% resulted in a much smaller increase in coverage accompanied with a relatively severe OP ratio, especially for the “identity ≤ 30%” group, indicating that 80% might be the optimal specificity threshold for DomSign. Thus, we applied this parameter in further analyses.

### Comparisons among DomSign, BLAST and FS models

BLAST was selected as the benchmark because of its wide application in research, and we used the best hit of BLAST to assign EC numbers. The FS model applies similar DS definitions, with no consideration for recurrence or order. However, it considers the contributions of every subset of DSs rather than regarding them as intact labels. Briefly, this model utilizes Bayesian statistical methods to evaluate the possibility of one particular GO annotation term inferred from all the subsets of the DS. By averaging the contributions of all the subsets, the probability of one protein having this annotation term can be calculated accordingly. There are three reasons for the comparison with the FS model: first, it utilizes domain information to assign GO terms. Thus, it can act as a good benchmark among the domain architecture–based methods. Secondly, this method yields reliable GO assignments, even in the situation where UniRef50 is applied for cross-validation, indicating the performance stability in an unbiased condition; and finally, the FS model provides a very user-friendly package for command line usage. Here, we converted GO terms to EC numbers using the EC2GO mapping file provided by the GO consortium [28].

Similar to the last section, to compare the performance among DomSign, BLAST and FS models, especially when the database contained no sequences having high similarities to the query protein, three rounds of 1,000-fold cross-validations (“identity ≤ 100%”, “identity ≤ 60%” and “identity ≤ 30%” as described in Methods) were conducted on the “sprot protein” dataset by DomSign with an 80% specificity threshold, BLASTP with a 10^−3^ E-value and the FS model with default parameter settings. It is necessary to emphasize the importance of performance tests using this scenario because BLAST itself performs enzyme functional annotations well (above 90% precision and recall in some situations) when homologs with similarities above a particular threshold are available [12]. Thus, there is limited room for further improvement in this regard, whereas there is ample need for improvement when homologs are unavailable. With the accumulation of novel sequences, this issue is expected to become more important. Thus, in the development of a new generation of computational approaches, more attention should be paid to the “homolog unavailable scenario”. As shown in Figure 3, machine learning–based methods, such as DomSign and the FS model, are much more robust when there is a reduced homolog availability compared with BLAST. Meanwhile, with a significant increase in “No best hit” (Figure 3B), coverage for BLAST decreases dramatically. Hence, in contrast to the nearly perfect performance of BLAST in the “identity ≤ 100%” group, DomSign achieved an overall performance superior to BLAST in the case of “identity ≤ 30%”, producing a comparable OP ratio but much higher coverage. Meanwhile, the FS model tended to have a very high OP ratio in all three tests, partly because of the multiple EC number predictions (Figure 3A) in this single EC enzyme plus non-enzyme dataset (Additional file 2) and partly because of incorrect EC assignments (both reasons contributed ~50% to the high OP level in the FS model, Figure 3A, B). Therefore, DomSign has the potential to partly replace BLAST as a functional annotation tool for novel proteins that have no homologs in the database.

### Comparison with SVMHL using an unbiased dataset

To further test the effectiveness of DomSign with respect to avoiding potential bias towards abundant enzyme families [32], the “SVMHL unbiased dataset” was subjected to a 10-fold cross-validation because any two sequences have <50% identity and the enzymes are manually selected to cover most of the enzyme families without bias. The SVMHL model [29] is the benchmark that annotates EC hierarchy by considering two main features, namely the abundance of every possible tripeptide sequence within a polypeptide [33] and a protein structure–based enzymatic function prediction model. The annotation accuracy of DomSign and SVMHL at the second and third EC hierarchy levels is shown in Additional file 5. Although the accuracy for the SVMHL model at the second hierarchy level was slightly greater than that of DomSign, at the third hierarchy level DomSign outperformed SVMHL for most enzyme families. Because Wang *et al.* [29] did not present their results at the fourth level, only the DomSign results at this level are shown (Additional file 5). Based on this comparison, DomSign works well in the unbiased situation compared with other benchmark methods.

### Comparison with EnzML

**Figure 4:**
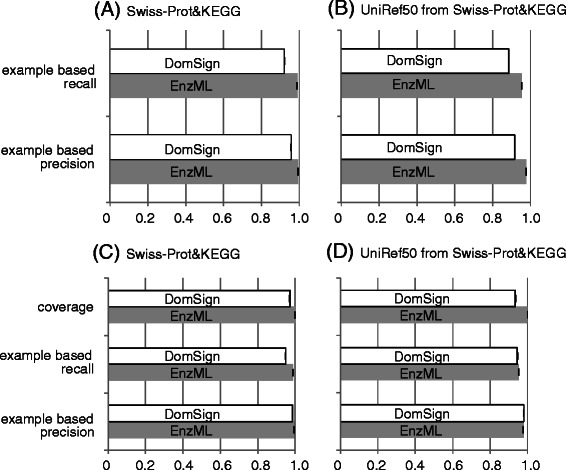
**Comparison between DomSign and EnzML using Swiss-Prot&KEGG and Swiss-Prot&KEGG extracted by UniRef50 datasets.** The barplot represents accuracy calucated by DomSign(white) and EnzML(gray). In contrast to panels **(A)** and **(B)**, enzymes that are incorrectly annotated as non-enzymes by DomSign are excluded from the evaluation in panels **(C)** and **(D)**. “Coverage” in panels **(C)** and **(D)** describes the percentage of proteins left after removal of real enzymes that were incorrectly predicted to be non-enzymes. ‘Example based precision’ and ‘Example based recall’ are used to evaluate the result as stated in Methods.

The EnzML model is a multi-label classification method that uses Binary Relevance Nearest Neighbors (BR-kNN) to predict EC numbers [30]. Briefly, this model utilized a more general protein signature set, InterPro [34], rather than Pfam as the input label. A multi-label support vector machine methodology was used, and the k parameter—the number of neighbors considered during the prediction—was optimized to ‘1’. The methodology of the multi-label support vector machines can be intuitively considered as the combination of multiple support vector machines for a series of binary labels (‘yes’ or ‘no’ for one particular EC hierarchy). Noteworthy, Mulan [35], an open-source software infrastructure for evaluation and prediction, is used for this specific work. This model is presently the best benchmark, which has been shown to be superior to some other widely used tools such as ModEnzA [36] and EFICAz2 [37]. “Swiss-Prot&KEGG” and the less redundant “UniRef50 Swiss-Prot&KEGG” [30] datasets were used for the 10-fold cross-validation (Figure 4A, B). Although the differences were not significant, we observed that EnzML performed better than DomSign in terms of example-based precision and recall. To clarify the source of these differences, for our evaluation we excluded the real enzymes that were incorrectly predicted as non-enzymes by DomSign (Figure 4C, D). Thereafter, DomSign’s performance became comparable to that of EnzML. Hence, we assert that the main reason for the loss of precision and recall in DomSign was that it is too strict to differentiate enzyme candidates from protein pools. Therefore, more enzymes are mistakenly categorized into the non-enzyme group by DomSign, leading to the loss of coverage. Even though this problem causes a decrease in the “example-based precision” defined here, it does not cause errors such as predicting the wrong EC number or mistakenly identifying a non-enzyme as an enzyme. Considering that the EnzML model is difficult to implement, we posit that using DomSign would be more facile by comparison with respect to expanding the enzyme space from a large-scale dataset, as discussed in the next section.

### Enzyme prediction in UniProt-TrEMBL and KEGG

Having demonstrated the reliability of DomSign, we annotated the whole protein space to determine if we could improve the prediction coverage of enzymes with EC numbers. UniProt-TrEMBL was used in this scenario owing to its exhaustive coverage of the known protein universe.

To test the precision of this enzyme prediction model, we ran the DomSign annotation against the “TrEMBL enzyme” set, which contained enzymes with single EC numbers in the TrEMBL database (Additional file 6). DomSign with an 80% specificity threshold yielded a 6.6% OP ratio while assigning EC numbers to ~90% enzymes. This OP ratio, which is higher than previous validations, may be due to the greater degree of error in the TrEMBL annotation [17]. This result, combined with the performance test, demonstrated that the enzyme space expansion effort we conducted, as described below, was highly reliable.

**Figure 5:**
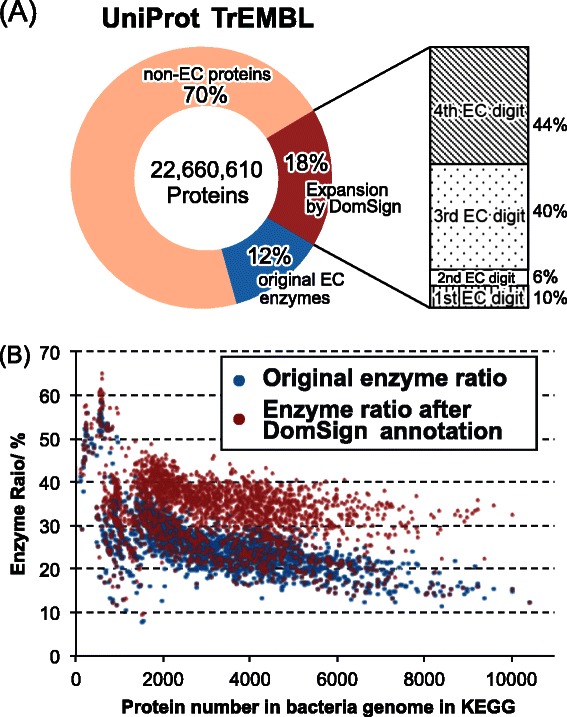
**Expansion of enzyme space in UniProt-TrEMBL and KEGG by DomSign (specificity threshold = 80%). (A)** Expansion of enzyme space in UniProt-TrEMBL. The circles illustrate the distribution of three kinds of proteins in the TrEMBL database. Blue: enzymes already tagged with EC numbers in TrEMBL; red: novel enzymes exclusively predicted by DomSign; light orange: other proteins without EC numbers. The column on the right represents the ratio of EC hierarchy levels among predicted novel enzymes by DomSign. Straight line: predicted enzymes annotated as EC = x.-.-.-; blank: annotated as EC = x.x.-.-; dot: annotated as EC = x.x.x.-; slash: annotated as EC = x.x.x.x. **(B)** Expansion of enzyme space in KEGG. Each blue dot represents the original enzyme ratio for one particular bacteria genome in KEGG. Each red dot represents the total enzyme ratio for one particular bacteria genome after DomSign annotation. In total, 2,584 bacterial genomes were tested.

Thus, we extended our data mining by predicting enzymes with EC numbers from all of the TrEMBL proteins. The annotation result is presented in Additional file 7. Approximately 3.9 million proteins lacking an EC number could be annotated with an EC number, and the majority of these belong to the three- or four-EC-digit group (Figure 5A). Even with a specificity threshold of 99%, the number of predicted novel enzymes was still around 3.6 million (Additional file 8), further indicating the reliability of this method. By this means, we successfully raised the EC-tagged enzyme ratio from the original 12% to ~30% in TrEMBL (Figure 5A) with high precision. To further illustrate the significance of this EC resource expansion, the increased EC-tagged enzyme ratios for every genome of the bacterial taxonomy in KEGG were calculated and are presented in Figure 5B (see Additional file 9 for detailed bacterial EC number annotations in KEGG). Remarkably, on average, we raised the EC-tagged enzyme ratio of each bacterial genome from the previous 26.0% to 33.2% for 2,584 bacterial genomes in KEGG, implying that the DomSign enzyme prediction method can provide deeper insight into the metabolism of many sequenced but insufficiently characterized organisms. Taken together, DomSign enzyme predictions in TrEMBL and KEGG increased the number of EC-labeled enzymes with precision and confirmed the existence of hypothetical gaps between the real enzyme space and the functional annotation.

### Enzyme predictions in metagenomic samples

Although millions of proteins have been discovered by the biological community, our knowledge of the protein world is still far from complete, and new metagenomic data provide us with new resources to explore [13]. Thus, we chose the HMP dataset as a test set to expand the enzyme space for proteins identified in metagenomic datasets using DomSign. Additionally, a combinational annotation pipeline in HMP using BLAST, TIGRFAM and Pfam-A [24] would be expected to be a good benchmark against which to compare DomSign in the functional annotations of metagenomic sequences.

As with TrEMBL, we first applied DomSign enzyme prediction to the “HMP enzyme” set to assess DomSign’s ability to predict enzymes. Compared with previous tests, much higher OP ratio (9.2%) was observed for DomSign with an 80% specificity threshold (Additional file 10). Despite the inability to evaluate the reliability of HMP annotations in this analysis, similar to the high error values in automatically annotated protein datasets such as TrEMBL [17], the quality of automatic HMP annotations is probably not as high as a manually curated set like Swiss-Prot. Thus, HMP annotation errors partly explain this abnormally high OP ratio, which is strongly supported by the fact that the OP ratio reached 5.4% even for DomSign with a 99% specificity threshold. These results still support the hypothesis that the reliability of the DomSign-based enzyme space expansion in HMP metagenomic datasets is acceptable.

**Figure 6:**
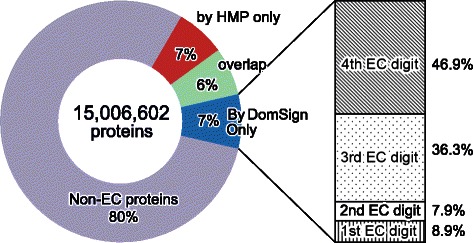
**Expansion of enzyme space in HMP non-redundant proteins by DomSign (specificity threshold = 80%).** The circles illustrate the distribution of four kinds of proteins in the HMP non-redundant dataset. Red: enzymes with EC numbers annotated exclusively by HMP; blue: novel enzymes exclusively predicted by DomSign; green: enzymes identified by both HMP and DomSign; purple: all remaining proteins. The column on the right represents the ratio of EC hierarchy levels for predicted novel enzymes by DomSign, similar to the description in Figure 5.

DomSign can recover more enzymes from this metagenomic dataset (Figure 6 and Additional file 11). Approximately one million new enzymes can be annotated with EC numbers exclusively by DomSign (around 7% of proteins in HMP set) (Additional file 12), and 84% of them contain at least three EC digits. DomSign and HMP also seem to be highly complementary because half of their identified enzymes do not overlap. This is probably owing to the low Pfam-A (45.7%) coverage of HMP proteins and the appearance of many novel DSs in metagenomic sequences. The complementary properties also indicate the possibility that DomSign can detect many different catalytic functions and thus may provide further insight into the metabolic capacity of the human microbiome. To test this hypothesis, we compared the unique four-digit EC numbers retrieved by both approaches. Here, the results for DomSign with a 99% specificity threshold were used to increase the reliability of EC number assignment. As an example, 81 novel EC numbers, which were exclusively detected by DomSign with a 99% specificity threshold, were discovered from the human gut microbiome (stool sample; Additional file 13), indicating one potential biologically significant discovery. These EC numbers may reflect important components that complement the known metabolism of the human microbiome.

## Discussion

### Limitations of DomSign

In this preliminary trial, our method performed well under diverse conditions, including having only distantly related sequences in the reference database (“sprot enzyme identity ≤ 30%”) and a query set without bias towards rich enzyme families (“SVMHL unbiased dataset”), indicating its potential to predict enzyme EC numbers in large-scale datasets. However, the precision and recall of this method are still not perfect.

First, even DomSign with a 99% specificity threshold results in a 3.6% OP ratio in the “identity ≤ 30%” 1,000-fold cross-validation. This is mainly because the domain architecture is unable to fully encode enzymatic activity, especially substrate specificity [38,39]. Substrate specificity determination is complex [40], especially for some superfamilies with diverse catalytic functions [41], and thus much effort has been devoted to this task using pioneering methods such as determining key functional residues in enzymes [42], key-residue 3D templates [43] and substrate *de novo* docking [44]. Future work will likely include the integration of these methodologies into our pipeline to more precisely predict the substrate specificity–determining fourth EC digit. With the development of DS databases, we can further increase the resolution of our method by involving more unique protein signatures, such as those from InterPro [34]. By this means, further increases in performance can be expected without changing the basic workflow of our method.

The comparison with SVMHL revealed variability in the performance of predicting EC number among different enzyme families. This corroborated a previous report that the worst result was obtained for oxidoreductase, as we observed with DomSign [30]. A possible solution is to utilize a combinational approach because different methodologies have diverse strengths for annotating specific enzyme families. SVMHL captures the sequence-function relationship of oxidoreductases quite well using triad abundance and structure [29]. Finally, as suggested by the comparison with EnzML, DomSign tends to have a high IA rate because it incorrectly predicts enzymes as non-enzymes. Considering that DomSign uses a very strict “yes or no” methodology to classify non-enzymes and enzymes at the first step in the pipeline, it could be improved by applying a probabilistic approach, such as the “specificity” we used in later iterations of DomSign for predicting EC numbers.

### Perspective expansion of enzyme space

To our knowledge, our present study represents the first systematic attempt to determine the extent to which the coverage of enzyme annotation by EC numbers could be improved, with acceptable precision, by methods beyond simple BLAST. By trying to close the gap between available EC-tagged enzymes in current databases and the real number of enzymes working in organisms, we showed that the quantity of EC-tagged enzymes can be significantly improved with high precision using relatively simple but reliable tools, such as DomSign, whether the sample is genomic or metagenomic. A series of assessments was performed to test the ability of DomSign to expand the enzyme space in large-scale protein datasets. This included a performance comparison with other benchmark enzyme annotation methods (Figures 3 and 4 Additional file 5) and a prediction and result comparison using large-scale protein sets whose members had already been assigned EC numbers, such as TrEMBL (Additional file 6) and HMP (Additional file 10). Under all conditions, the precision rate was >90% and recall was quite remarkable.

The results of the first large-scale critical assessment of protein function annotations (CAFA) were recently published [7]. One of the main conclusions of CAFA was that many advanced methods for protein function annotation are superior to the first generation of methods, such as BLAST. Most of the top-ranked methods in CAFA utilized a machine learning–based computational approach. As suggested by Furnham N *et al.* [10], however, first-generation annotation methods are still used in most research. For instance, in a previous version of SEED, an intensively used comparative genomics environment, homology-based functional transfer is the main method of annotation. This is also true for UniProt. In recent releases, UniProt incorporated the HAMAP system [45], and SEED complements its annotation strategy using a k-mer-based subsystem and FIGfam recognition approach [46]; still, these approaches depend on sequence similarity–based function transfers, such as functionally homologous family profiles. The situation is essentially the same for benchmark metagenomic projects such as HMP [24,47]. With the development of metagenomics, many more sequences will be derived from environmental samples and will be novel compared with the current databases. In such cases, as shown in our work and that of many others [11,13], similarity-based function transfer will struggle to achieve the desired performance.

As our work demonstrates, there is still need to improve the ability to predict more enzymes using *in silico* methods. Only 12% of the proteins in UniProt have EC numbers. In the HMP phase I 95% non-redundant set, this value is 13% (Figure 6). All of the values are far below the average 30% enzyme ratio of the nine intensively studied organisms [48]. We believe that a richer annotated sequence resource will result once this gap is closed using a hierarchical or top-down machine-learning method. This will allow researchers to not only study many important biological questions such as orphan enzyme gene identification [49] and metabolism network reconstruction [50] but also improve strategies used in biotechnology, including secondary metabolism gene cluster identification [51], artificial biosynthesis pathway design [52], novel enzyme mining [53] and metabolic engineering [54].

## Conclusions

In this work, we developed a novel enzyme EC number prediction tool, DomSign, which is superior to conventional BLAST for the homolog unavailable scenario. In addition, other novel and outstanding enzyme functional annotation tools were selected as benchmarks and these were used to run comparisons against DomSign, which confirmed the superior or competitive ability in enzyme functional annotation of DomSign. The DomSign method requires only the amino acid sequences, without the need for existing annotations or structures. Based on the test results, the performance of DomSign should be improved by incorporating more exhaustive protein signatures, such as substrate specificity-determining residues, and revising the pipeline to select enzyme candidates using a probabilistic approach.

Using DomSign, we tried to address whether a large number of ‘hidden enzymes’ without EC number annotations exist in current protein databases, such as TrEMBL, KEGG and metagenomic sets like HMP. Our results preliminarily confirmed this hypothesis by significantly improving the ratio of EC-tagged enzymes in these databases. The illustration and annotation of these enzymes should significantly deepen our understanding of the metabolisms of diverse organisms or consortia, and also facilitate bioengineering by providing a richer enzyme resource. Furthermore, our results highlight the necessity to involve more advanced tools than BLAST in protein database annotations, thereby extracting more biological information from the available number of biological sequences.

## Additional files

Additional file 1:
**Domain Signature (DS)-EC pair specificity distribution in DomSign EC prediction model and comparison between considering order, recurrence or not.** This dataset shows the structure of machine learning model while considering the domain recurrence and order or not. Briefly, different definitions about protein domain signature (considering the domain recurrence and order or not) are applied to construct the machine learning model for EC number prediction as suggested in Figure 1 with ‘sprot enzyme’ (mentioned in Additional file 13 and ‘Method’ section) as training set. For each model, a series of DS-EC pairs are constructed with a defined ‘specificity’. The distribution of ‘specificity’ of these DS-EC pairs are represented in this figure. The order and recurrence information of Pfam-A domain is extracted from swisspfam.gz dataset from Pfam FTP site (ftp://ftp.ebi.ac.uk/pub/databases/Pfam/current_release/swisspfam.gz).
Additional file 2:
**Datasets used in this work.** Summary of the datasets used in this work including dataset name, processing description, enzyme/non-enzyme ratio, unique domain signature number and total entry number for each set.
Additional file 3:
**DomSign tool developed in this work.** The compressed directory contains the shell and python scripts of DomSign used in this work. It can be used as a stand-alone tool in linux environment. Both EC number prediction and cross-validation functions are embed in this tool for automatic processing. It thus can be used to reproduce all the results we have presented in the paper or act as basis for other machine learning approach in EC number prediction. For details about the tool usage, please check the README file in the decompressed directory.
Additional file 4:
**DomSign specificity threshold optimization by 1000-fold cross validation on “Sprot protein” (53.2% non-enzyme in contrast to 46.8% enzyme).** In this test, 99%, 90%, 80% and 70% specificity thresholds were applied in DomSign to test its influence on the performance of DomSign. Here, three kinds of 1000-fold cross validations are conducted for each methods. For each kind of cross-validation, homologous sequences of query above a given threshold (“identity ≤ 100%”, “identity ≤ 60%” and “identity ≤ 30%” as described in Methods) in reference dataset are removed to simulate the situation where there are no sequences with high similarity towards query proteinin available database. Thereafter, for each reference dataset, only sequences below the given threshold are kept, corresponding to the “identity ≤ 100%, 60% and 30%” in the figure, respectively. All the relative standard errors are not significant (<1%) thus not illustrated here. (A) Result evaluation of different methods. As shown on the right part, four attributes are defined to describe the annotation result in contrast to the “true EC number”. For details, please see Methods. (B) EC hierarchy level distribution of annotation result for different methods.
Additional file 5:
**Performance comparison between DomSign (80% specificity threshold) and SVMHL against one unbiased dataset.** All the error bars presented here are standard error by 10-fold cross validation. (a) The prediction accuracy at the second level of EC hierarchy for SVMHL and DomSign. White column: SVMHL; Grey column: DomSign. (b) The prediction accuracy at the third level of EC hierarchy for SVMHL and DomSign. White column: SVMHL; Grey column: DomSign. (c) The prediction accuracy at the second, third and fourth level of EC hierarchy for DomSign. White, light grey and dark grey columns correspond to the second, third and fourth level of EC hierarchy, respectively.
Additional file 6:
**Enzymes with single EC number in UniProt-TrEMBL (“trembl enzyme” mentioned in** Methods **)** **annotated by DomSign with different specificity thresholds (99%, 90%, 80% and 70%).** The result evaluation and illustration method is similar to that described in Additional file 4. (A) Result evaluation by DomSign with different specificity thresholds. (B) EC hierarchy level distribution in annotation result by DomSign with different specificity thresholds.
Additional file 7:
**Enzymes from TrEMBL annotated with at least three EC digit by DomSign.** This dataset contains all the enzymes in TrEMBL database and their relevant EC numbers designated by DomSign in tab separated format. To limit the size of the file, only enzymes assigned with at least three EC digit are extracted (84% of all the predicted enzymes by DomSign). All the protein accession numbers are UniProt protein ID.
Additional file 8:
**Predicted novel enzymes from TrEMBL by DomSign with different specificity thresholds (99%, 90%, 80%, 70%).** The stacked columns represent the ratio of EC hierarchy levels assigned by DomSign. Straight Line: predicted enzymes annotated as E.C. = x.-.-.- (1st E.C. digit), Blank: annotated as E.C. = x.x.-.- (2nd E.C. digit), Dot: annotated as E.C. = x.x.x.- (3rd E.C. digit), Slash: annotated as E.C. = x.x.x.x (4th E.C. digit). Black solid line refers to absolute number of predicted novel enzymes by DomSign supervised with different specificity thresholds.
Additional file 9:
**Novel enzymes identified by DomSign from KEGG database bacteria subsection.** This dataset contains all the novel enzymes in KEGG database bacteria subsection and their relevant EC numbers designated by DomSign in tab separated format. Only enzymes uniquely identified by DomSign beyond KEGG enzyme flat file are extracted, which increase the genomic enzyme ratio in KEGG bacteria from original 26% to 33% on average. All the protein accession numbers are KEGG gene ID.
Additional file 10:
**Enzymes with single EC numbers in HMP phase I non-redundant dataset (“HMP enzyme” described in** Methods **)** **annotated by DomSign with different specificity thresholds (99%, 90%, 80% and 70%).** The result evaluation and illustration method is similar to that described in Additional file 4 and Additional file 6. (A) Result evaluation by DomSign with different specificity thresholds. (B) EC hierarchy level distribution of annotation result by DomSign with different specificity thresholds.
Additional file 11:
**Enzymes from HMP phase I 95% non-redundant dataset identified by DomSign.** This dataset contains all the enzymes and their relevant EC numbers in HMP phase I 95% non-redundant dataset designated by DomSign in tab separated format. All the protein accession numbers are extracted from HMP DACC raw dataset for phase I 95% non-redundant predicted genes
Additional file 12:
**Enzyme prediction from HMP phase I non-redundant proteins with different specificity thresholds (99%, 90%, 80% and 70%).** Stacked columns divided into different patterns refer to different EC hierarchy evels in annotation result as described in Additional file 8. The performance of DomSign with 99%, 90%, 80% and 70% specificity thresholds are compared with original HMP annotation result. HMP-PfamA-protein refers to the enzyme subset of HMP non-redundant proteins encompassing Pfam-A domains.
Additional file 13:
**81 novel EC numbers detected by DomSign.** DomSign (99% specificity threshold) can extract 81 novel four-digit EC numbers from Human Microbiome Project gut subset beyond the HMP official annotation. The EC numbers and their corresponding enzymatic reactions extracted from KEGG database are listed.

## References

[1] Friedberg I (2006). Automated protein function prediction–the genomic challenge. Brief Bioinform.

[2] Pitkänen E, Rousu J, Ukkonen E (2010). Computational methods for metabolic reconstruction. Curr Opin Biotechnol.

[3] Roy A, Yang J, Zhang Y (2012). COFACTOR: an accurate comparative algorithm for structure-based protein function annotation. Nucleic Acids Res.

[4] Lee DA, Rentzsch R, Orengo C (2010). GeMMA: functional subfamily classification within superfamilies of predicted protein structural domains. Nucleic Acids Res.

[5] Gaudet P, Livstone MS, Lewis SE, Thomas PD (2011). Phylogenetic-based propagation of functional annotations within the Gene Ontology consortium. Brief Bioinform.

[6] Jensen LJ, Kuhn M, Stark M, Chaffron S, Creevey C, Muller J (2009). STRING 8–a global view on proteins and their functional interactions in 630 organisms. Nucleic Acids Res.

[7] Radivojac P, Clark WT, Oron TR, Schnoes AM, Wittkop T, Sokolov A (2013). A large-scale evaluation of computational protein function prediction. Nat Methods.

[8] Yu C, Zavaljevski N, Desai V, Reifman J (2009). Genome-wide enzyme annotation with precision control: catalytic families (CatFam) databases. Proteins.

[9] Altschul SF, Madden TL, Schäffer AA, Zhang J, Zhang Z, Miller W (1997). Gapped BLAST and PSI-BLAST : a new generation of protein database search programs. Nucleic Acids Res.

[10] Furnham N, Garavelli JS, Apweiler R, Thornton JM (2009). Missing in action: enzyme functional annotations in biological databases. Nat Chem Biol.

[11] Rost B (2002). Enzyme function less conserved than anticipated. J Mol Biol.

[12] Addou S, Rentzsch R, Lee D, Orengo CA (2009). Domain-based and family-specific sequence identity thresholds increase the levels of reliable protein function transfer. J Mol Biol.

[13] Hess M, Sczyrba A, Egan R, Kim T-W, Chokhawala H, Schroth G (2011). Metagenomic discovery of biomass-degrading genes and genomes from cow rumen. Science.

[14] Todd AE, Orengo CA, Thornton JM (2001). Evolution of function in protein superfamilies, from a structural perspective. J Mol Biol.

[15] Shen H-B, Chou K-C (2007). EzyPred: a top-down approach for predicting enzyme functional classes and subclasses. Biochem Biophys Res Commun.

[16] Akiva E, Brown S, Almonacid DE, Barber AE, Custer AF, Hicks MA (2014). The structure-function linkage database. Nucleic Acids Res.

[17] Schnoes AM, Brown SD, Dodevski I, Babbitt PC (2009). Annotation error in public databases: misannotation of molecular function in enzyme superfamilies. PLoS Comput Biol.

[18] Ashburner M, Ball CA, Blake JA, Botstein D, Butler H, Cherry JM (2000). Gene ontology: tool for the unification of biology. The gene ontology consortium. Nat Genet.

[19] Forslund K, Sonnhammer ELL (2008). Predicting protein function from domain content. Bioinformatics.

[20] Fang H, Gough J (2013). DcGO: database of domain-centric ontologies on functions, phenotypes, diseases and more. Nucleic Acids Res.

[21] Rentzsch R, Orengo CA (2009). Protein function prediction–the power of multiplicity. Trends Biotechnol.

[22] The UniProt Consortium (2014). Activities at the Universal Protein Resource (UniProt). Nucleic Acids Res.

[23] Kanehisa M (2000). KEGG: kyoto encyclopedia of genes and genomes. Nucleic Acids Res.

[24] The Human Microbiome Project Consortium (2012). A framework for human microbiome research. Nature.

[25] Punta M, Coggill PC, Eberhardt RY, Mistry J, Tate J, Boursnell C (2012). The Pfam protein families database. Nucleic Acids Res.

[26] Messih MA, Chitale M, Bajic VB, Kihara D, Gao X (2012). Protein domain recurrence and order can enhance prediction of protein functions. Bioinformatics.

[27] Eddy SR (2011). Accelerated profile HMM searches. PLoS Comput Biol.

[28] Hill DP, Davis AP, Richardson JE, Corradi JP, Ringwald M, Eppig JT (2001). Program description: strategies for biological annotation of mammalian systems: implementing gene ontologies in mouse genome informatics. Genomics.

[29] Wang Y-C, Wang Y, Yang Z-X, Deng N-Y (2011). Support vector machine prediction of enzyme function with conjoint triad feature and hierarchical context. BMC Syst Biol.

[30] De Ferrari L, Aitken S, van Hemert J, Goryanin I (2012). EnzML: multi-label prediction of enzyme classes using InterPro signatures. BMC Bioinformatics.

[31] Tsoumakas G, Katakis I, Vlahavas I: Data Mining and Knowledge Discovery Handbook. 2010(Mlc).

[32] Chou K-C (2011). Some remarks on protein attribute prediction and pseudo amino acid composition. J Theor Biol.

[33] Shen J, Zhang J, Luo X, Zhu W, Yu K, Chen K (2007). Predicting protein-protein interactions based only on sequences information. Proc Natl Acad Sci U S A.

[34] Hunter S, Apweiler R, Attwood TK, Bairoch A, Bateman A, Binns D (2009). InterPro: the integrative protein signature database. Nucleic Acids Res.

[35] Tsoumakas G, Spyromitros-Xioufis E, Vilcek J, Vlahavas I (2011). MULAN: a java library for multi-label learning. J Mach Learn Res.

[36] Desai DK, Nandi S, Srivastava PK, Lynn AM (2011). ModEnzA: accurate identification of metabolic enzymes using function specific profile HMMs with optimised discrimination threshold and modified emission probabilities. Adv Bioinformatics.

[37] Kumar N, Skolnick J (2012). EFICAz2.5: application of a high-precision enzyme function predictor to 396 proteomes. Bioinformatics.

[38] Bashton M, Thornton JM (2009). Domain-ligand mapping for enzymes. J Mol Recognit.

[39] Brown SD, Gerlt JA, Seffernick JL, Babbitt PC (2006). A gold standard set of mechanistically diverse enzyme superfamilies. Genome Biol.

[40] Rodriguez GJ, Yao R, Lichtarge O, Wensel TG (2010). Evolution-guided discovery and recoding of allosteric pathway specificity determinants in psychoactive bioamine receptors. Proc Natl Acad Sci U S A.

[41] Nagao C, Nagano N, Mizuguchi K (2010). Relationships between functional subclasses and information contained in active-site and ligand-binding residues in diverse superfamilies. Proteins.

[42] Arakaki AK, Huang Y, Skolnick J (2009). EFICAz2: enzyme function inference by a combined approach enhanced by machine learning. BMC Bioinformatics.

[43] Amin SR, Erdin S, Ward RM, Lua RC, Lichtarge O (2013). Prediction and experimental validation of enzyme substrate specificity in protein structures. Proc Natl Acad Sci U S A.

[44] Zhao S, Kumar R, Sakai A, Vetting MW, Wood BM, Brown S (2013). Discovery of new enzymes and metabolic pathways by using structure and genome context. Nature.

[45] Pedruzzi I, Rivoire C, Auchincloss AH, Coudert E, Keller G, de Castro E (2013). HAMAP in 2013, new developments in the protein family classification and annotation system. Nucleic Acids Res.

[46] Overbeek R, Olson R, Pusch GD, Olsen GJ, Davis JJ, Disz T (2014). The SEED and the rapid annotation of microbial genomes using subsystems technology (RAST). Nucleic Acids Res.

[47] Tanenbaum DM, Goll J, Murphy S, Kumar P, Zafar N, Thiagarajan M (2010). The JCVI standard operating procedure for annotating prokaryotic metagenomic shotgun sequencing data. Stand Genomic Sci.

[48] Quester S, Schomburg D (2011). EnzymeDetector: an integrated enzyme function prediction tool and database. BMC Bioinformatics.

[49] Yamada T, Waller AS, Raes J, Zelezniak A, Perchat N, Perret A (2012). Prediction and identification of sequences coding for orphan enzymes using genomic and metagenomic neighbours. Mol Syst Biol.

[50] Orth JD, Conrad TM, Na J, Lerman JA, Nam H, Feist AM (2011). A comprehensive genome-scale reconstruction of Escherichia coli metabolism–2011. Mol Syst Biol.

[51] Medema MH, Blin K, Cimermancic P, De Jager V, Zakrzewski P, Fischbach MA (2011). AntiSMASH: rapid identification, annotation and analysis of secondary metabolite biosynthesis gene clusters in bacterial and fungal genome sequences. Nucleic Acids Res.

[52] Carbonell P, Parutto P, Herisson J, Pandit SB, Faulon J-L (2014). XTMS: pathway design in an eXTended metabolic space. Nucleic Acids Res.

[53] Schallmey M, Koopmeiners J, Wells E, Wardenga R, Schallmey A (2014). Expanding the halohydrin dehalogenase enzyme family: identification of novel enzymes by database mining. Appl Environ Microbiol.

[54] Ro D-K, Paradise EM, Ouellet M, Fisher KJ, Newman KL, Ndungu JM (2006). Production of the antimalarial drug precursor artemisinic acid in engineered yeast. Nature.

