# Supplementary material for: DomSign: a top-down annotation pipeline to enlarge enzyme space in the protein universe
Source: BMC Bioinformatics. 2015 Mar 21;16:96. doi: 10.1186/s12859-015-0499-y (PMC4389672; doi:10.1186/s12859-015-0499-y)
Supplement: Additional file 1: — Domain Signature (DS)-EC pair specificity distribution in DomSign EC prediction model and comparison between considering order, recurrence or not. This dataset shows the structure of machine learning model while considering the domain recurrence and order or not. Briefly, different definitions about protein domain signature (considering the domain recurrence and order or not) are applied to construct the machine learning model for EC number prediction as suggested in Figure 1 with ‘sprot enzyme’ (mentioned in Additional file 13 and ‘Method’ section) as training set. For each model, a series of DS-EC pairs are constructed with a defined ‘specificity’. The distribution of ‘specificity’ of these DS-EC pairs are represented in this figure. The order and recurrence information of Pfam-A domain is extracted from swisspfam.gz dataset from Pfam FTP site (ftp://ftp.ebi.ac.uk/pub/databases/Pfam/current_release/swisspfam.gz). [file 12859_2015_499_MOESM1_ESM.pdf]

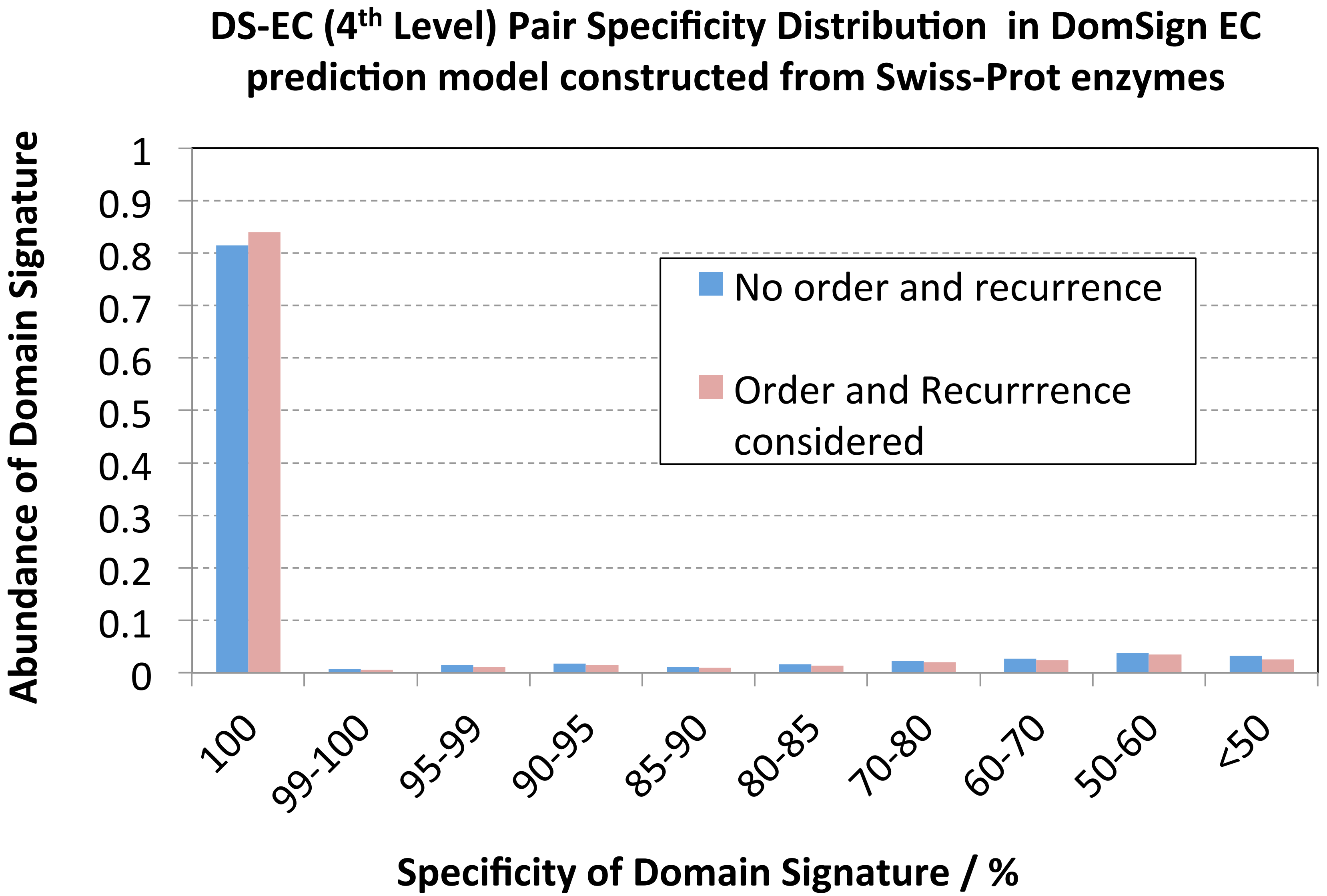

DS-EC (4th Level) Pair Specificity Distribution in DomSign EC prediction model constructed from Swiss-Prot enzymes
Abundance of Domain Signature
1
0.9
0.8
0.7
0.6
0.5
0.4
0.3
0.2
0.1
0
No order and recurrence
Order and Recurrrence considered
100
99-100
95-99
90-95
85-90
80-85
70-80
60-70
50-60
<50
Specificity of Domain Signature / %
