# Supplementary figures and images for: DomSign: a top-down annotation pipeline to enlarge enzyme space in the protein universe
Source: BMC Bioinformatics. 2015 Mar 21;16:96. doi: 10.1186/s12859-015-0499-y (PMC4389672; doi:10.1186/s12859-015-0499-y)

(A)

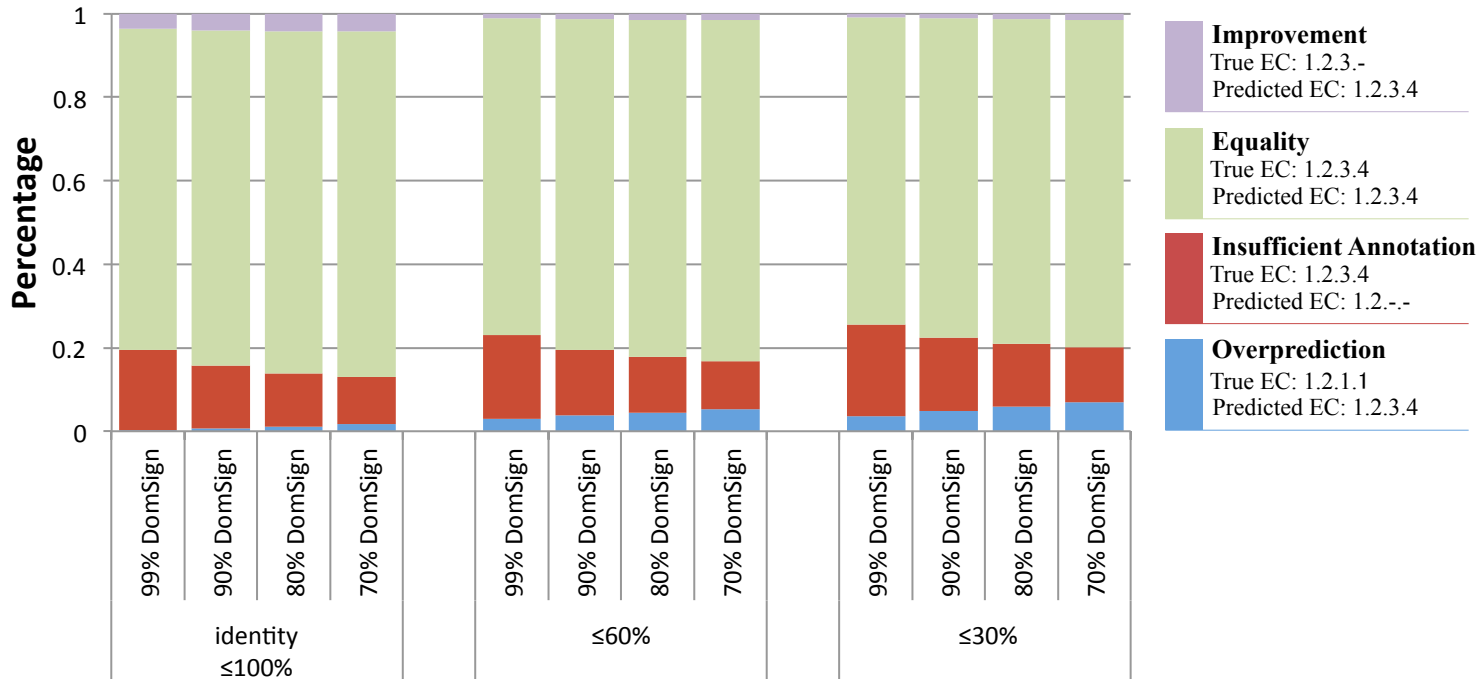

(B)

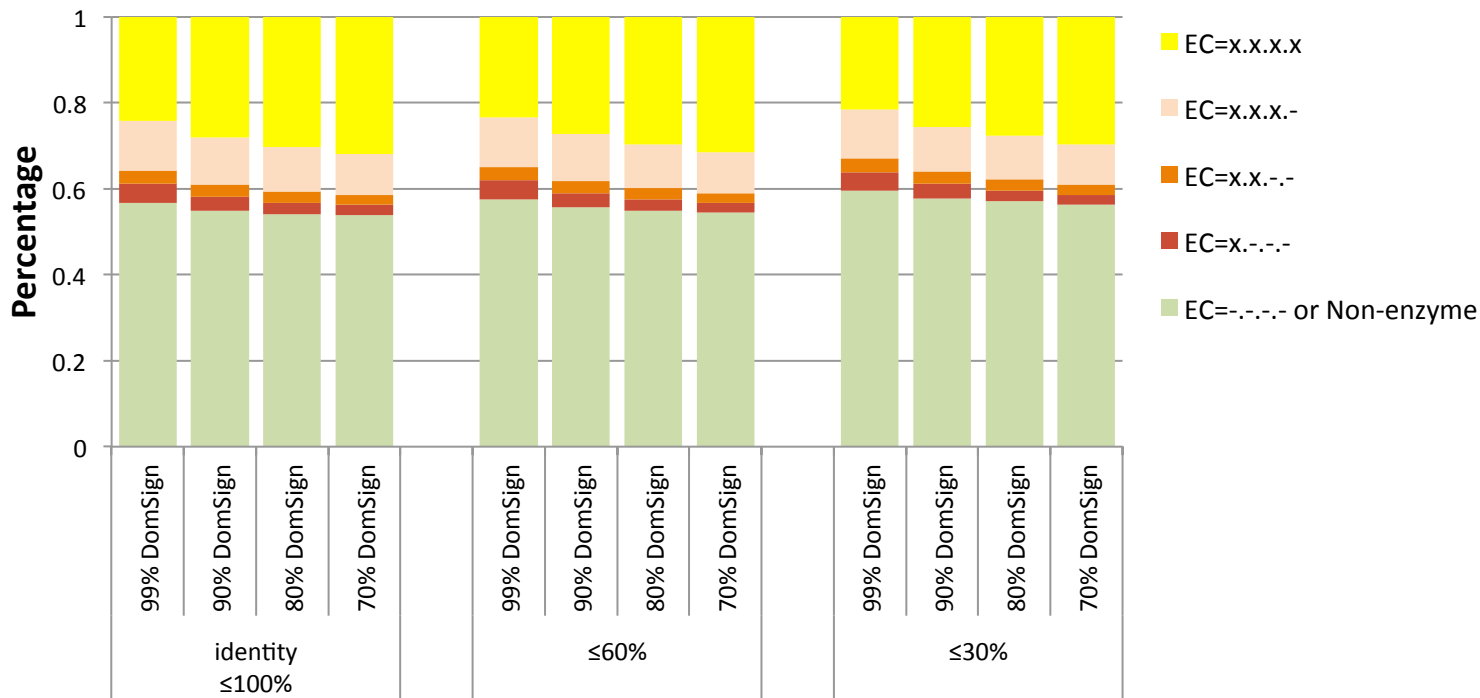

Supplement: Additional file 4: — DomSign specificity threshold optimization by 1000-fold cross validation on “Sprot protein” (53.2% non-enzyme in contrast to 46.8% enzyme). In this test, 99%, 90%, 80% and 70% specificity thresholds were applied in DomSign to test its influence on the performance of DomSign. Here, three kinds of 1000-fold cross validations are conducted for each methods. For each kind of cross-validation, homologous sequences of query above a given threshold (“identity ≤ 100%”, “identity ≤ 60%” and “identity ≤ 30%” as described in Methods) in reference dataset are removed to simulate the situation where there are no sequences with high similarity towards query proteinin available database. Thereafter, for each reference dataset, only sequences below the given threshold are kept, corresponding to the “identity ≤ 100%, 60% and 30%” in the figure, respectively. All the relative standard errors are not significant (<1%) thus not illustrated here. (A) Result evaluation of different methods. As shown on the right part, four attributes are defined to describe the annotation result in contrast to the “true EC number”. For details, please see Methods. (B) EC hierarchy level distribution of annotation result for different methods. [file 12859_2015_499_MOESM4_ESM.pdf]

(A)

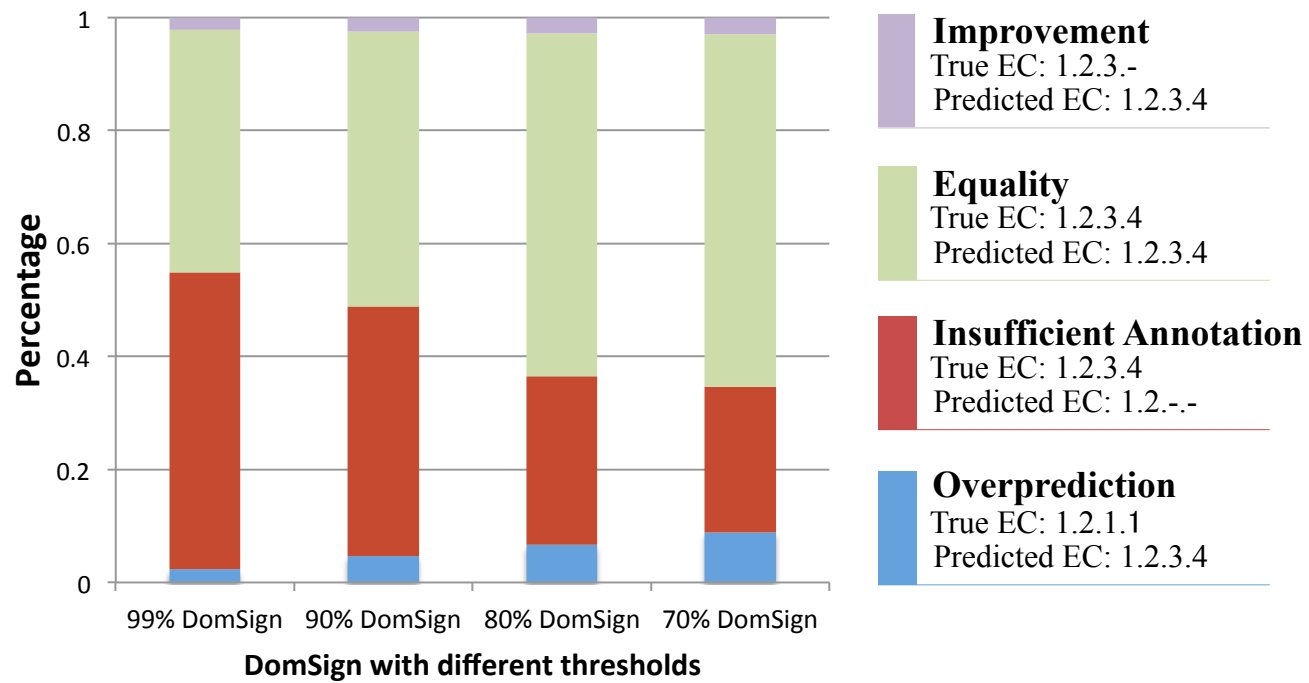

(B)

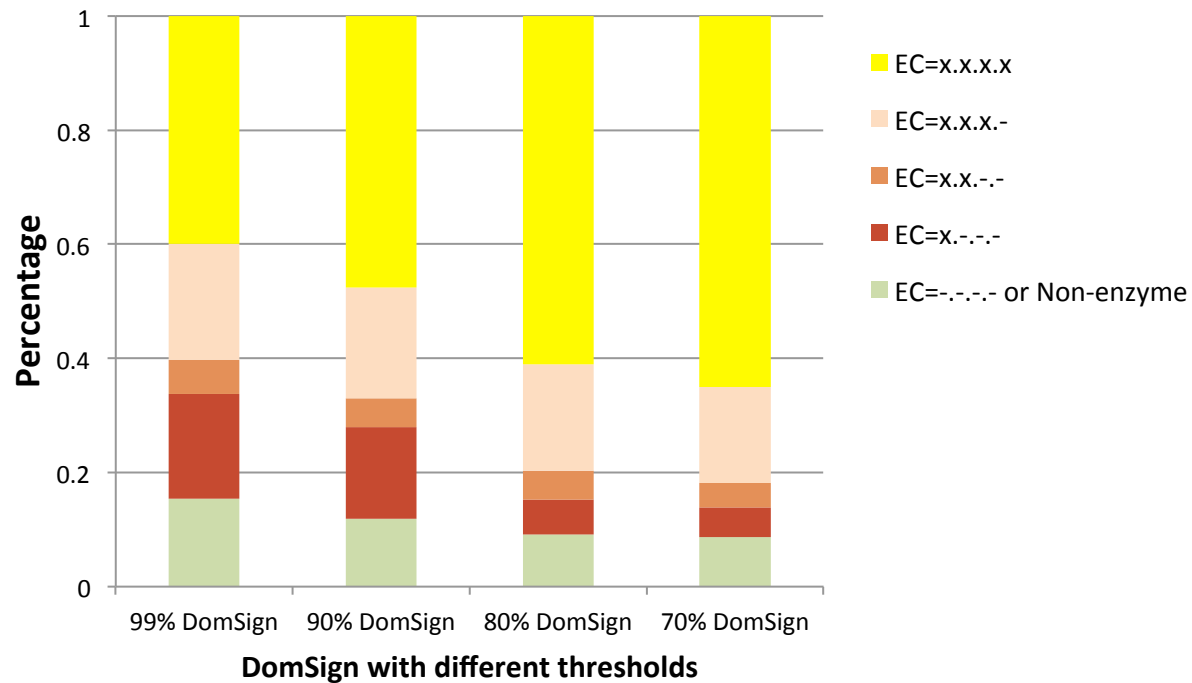

Supplement: Additional file 6: — Enzymes with single EC number in UniProt-TrEMBL (“trembl enzyme” mentioned in Methods ) annotated by DomSign with different specificity thresholds (99%, 90%, 80% and 70%). The result evaluation and illustration method is similar to that described in Additional file 4. (A) Result evaluation by DomSign with different specificity thresholds. (B) EC hierarchy level distribution in annotation result by DomSign with different specificity thresholds. [file 12859_2015_499_MOESM6_ESM.pdf]

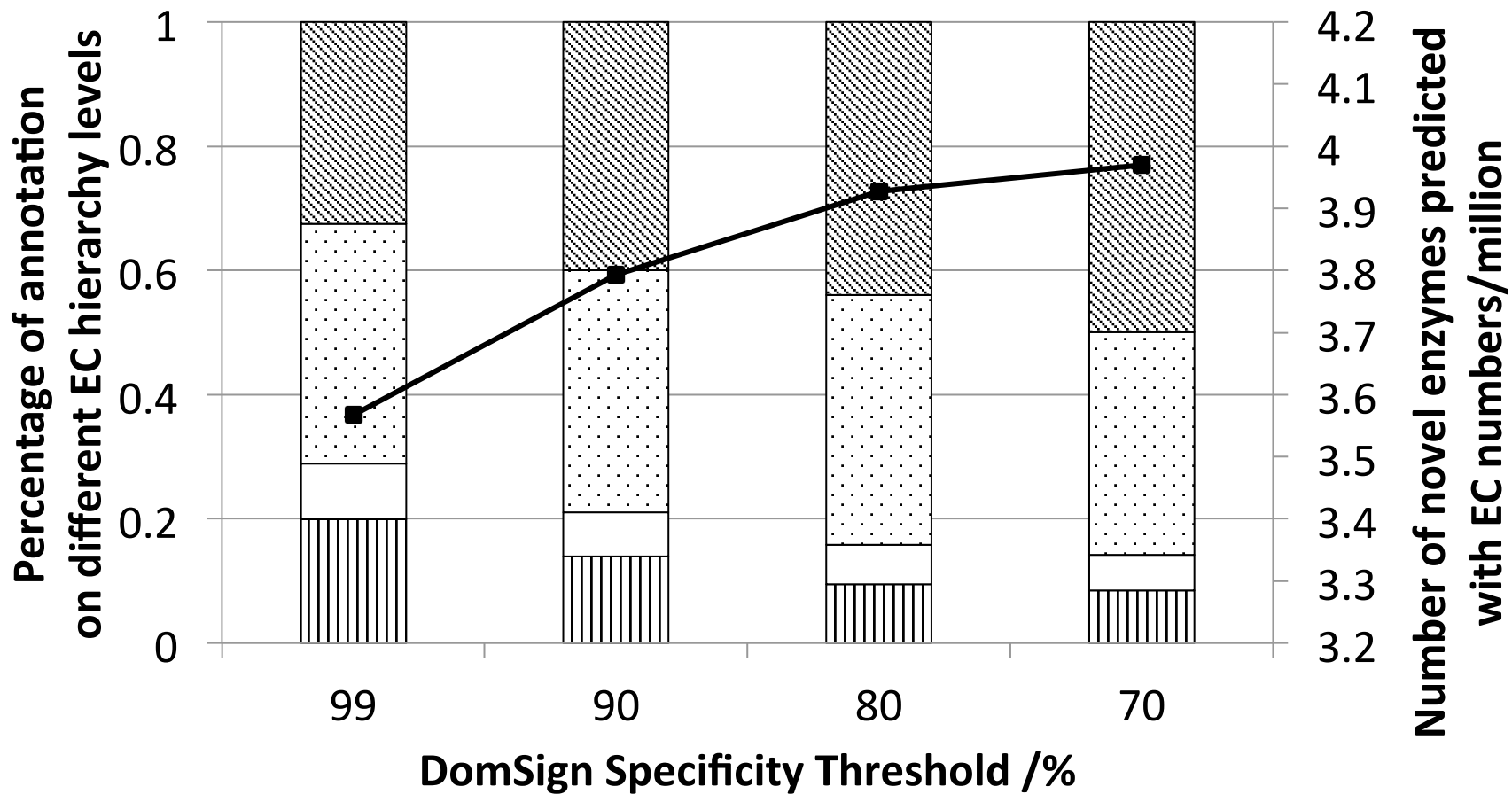

1st 2nd 3rd 4th ■ novel enzymes predicted

Supplement: Additional file 8: — Predicted novel enzymes from TrEMBL by DomSign with different specificity thresholds (99%, 90%, 80%, 70%). The stacked columns represent the ratio of EC hierarchy levels assigned by DomSign. Straight Line: predicted enzymes annotated as E.C. = x.-.-.- (1st E.C. digit), Blank: annotated as E.C. = x.x.-.- (2nd E.C. digit), Dot: annotated as E.C. = x.x.x.- (3rd E.C. digit), Slash: annotated as E.C. = x.x.x.x (4th E.C. digit). Black solid line refers to absolute number of predicted novel enzymes by DomSign supervised with different specificity thresholds. [file 12859_2015_499_MOESM8_ESM.pdf]

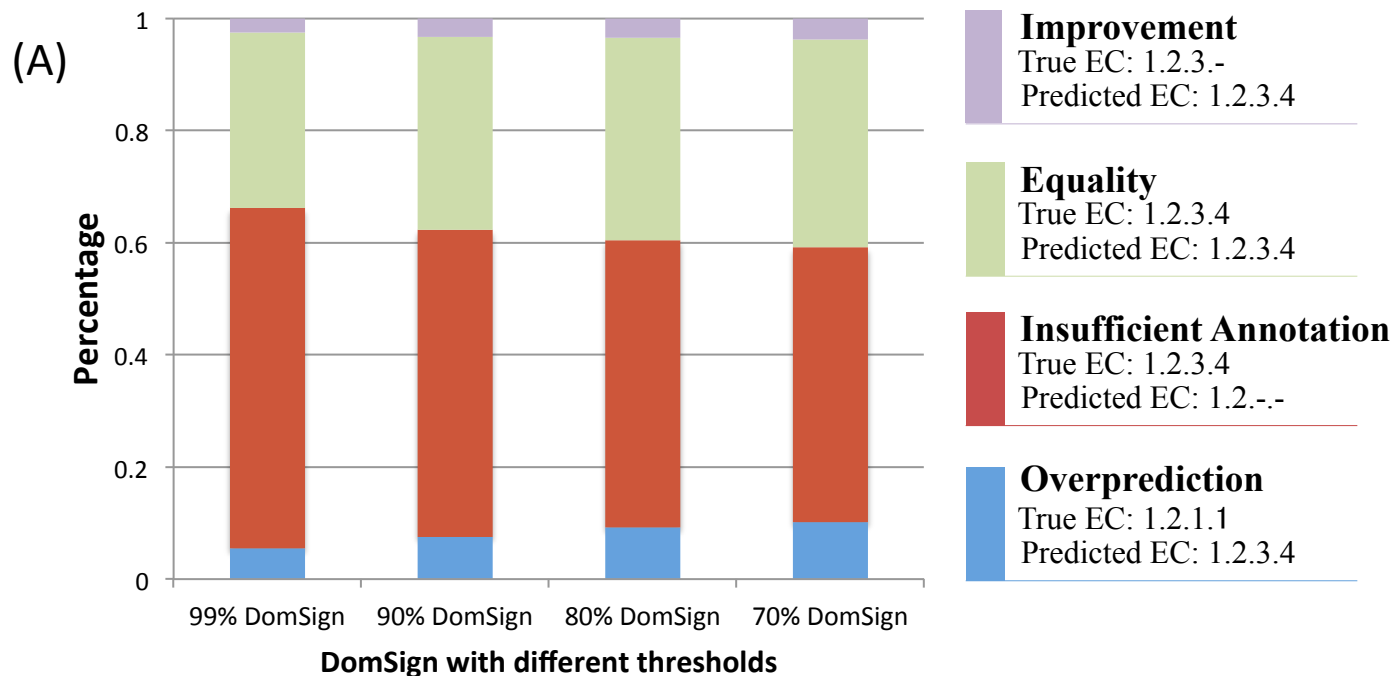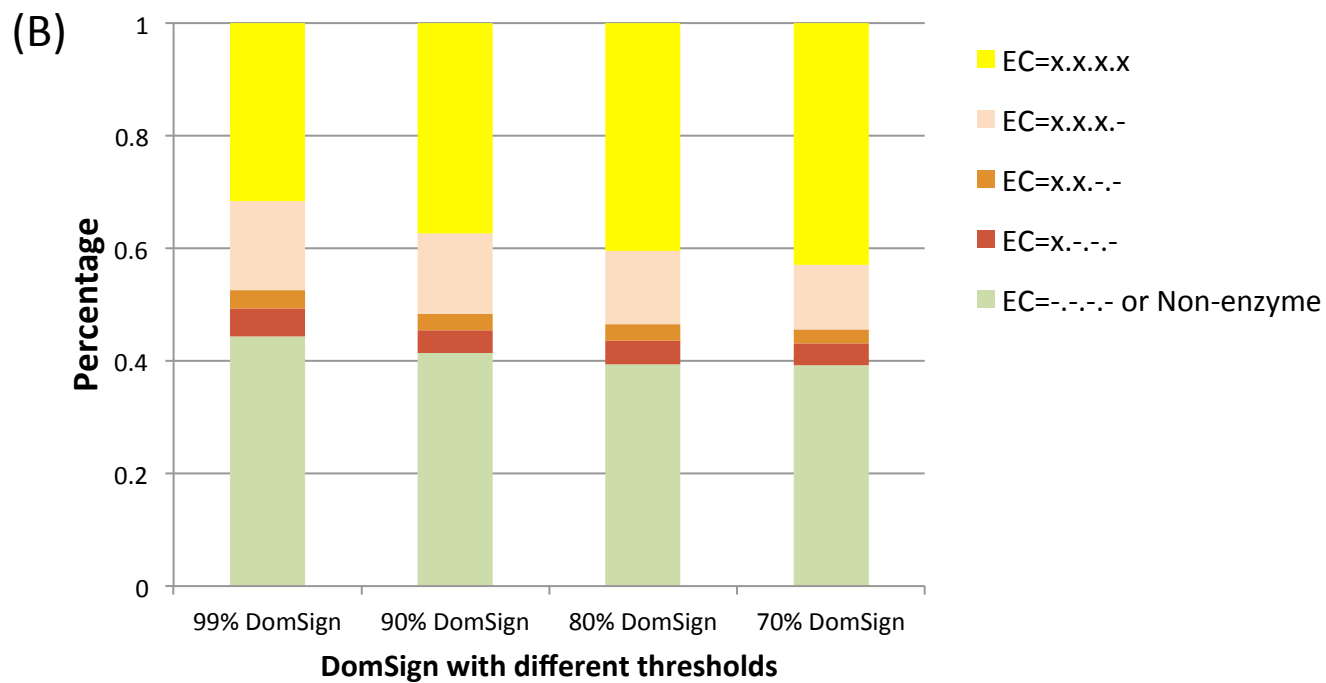

Supplement: Additional file 10: — Enzymes with single EC numbers in HMP phase I non-redundant dataset (“HMP enzyme” described in Methods ) annotated by DomSign with different specificity thresholds (99%, 90%, 80% and 70%). The result evaluation and illustration method is similar to that described in Additional file 4 and Additional file 6. (A) Result evaluation by DomSign with different specificity thresholds. (B) EC hierarchy level distribution of annotation result by DomSign with different specificity thresholds. [file 12859_2015_499_MOESM10_ESM.pdf]
