# Supplementary material for: DomSign: a top-down annotation pipeline to enlarge enzyme space in the protein universe
Source: BMC Bioinformatics. 2015 Mar 21;16:96. doi: 10.1186/s12859-015-0499-y (PMC4389672; doi:10.1186/s12859-015-0499-y)
Supplement: Additional file 5: — Performance comparison between DomSign (80% specificity threshold) and SVMHL against one unbiased dataset. All the error bars presented here are standard error by 10-fold cross validation. (a) The prediction accuracy at the second level of EC hierarchy for SVMHL and DomSign. White column: SVMHL; Grey column: DomSign. (b) The prediction accuracy at the third level of EC hierarchy for SVMHL and DomSign. White column: SVMHL; Grey column: DomSign. (c) The prediction accuracy at the second, third and fourth level of EC hierarchy for DomSign. White, light grey and dark grey columns correspond to the second, third and fourth level of EC hierarchy, respectively. [file 12859_2015_499_MOESM5_ESM.pdf]

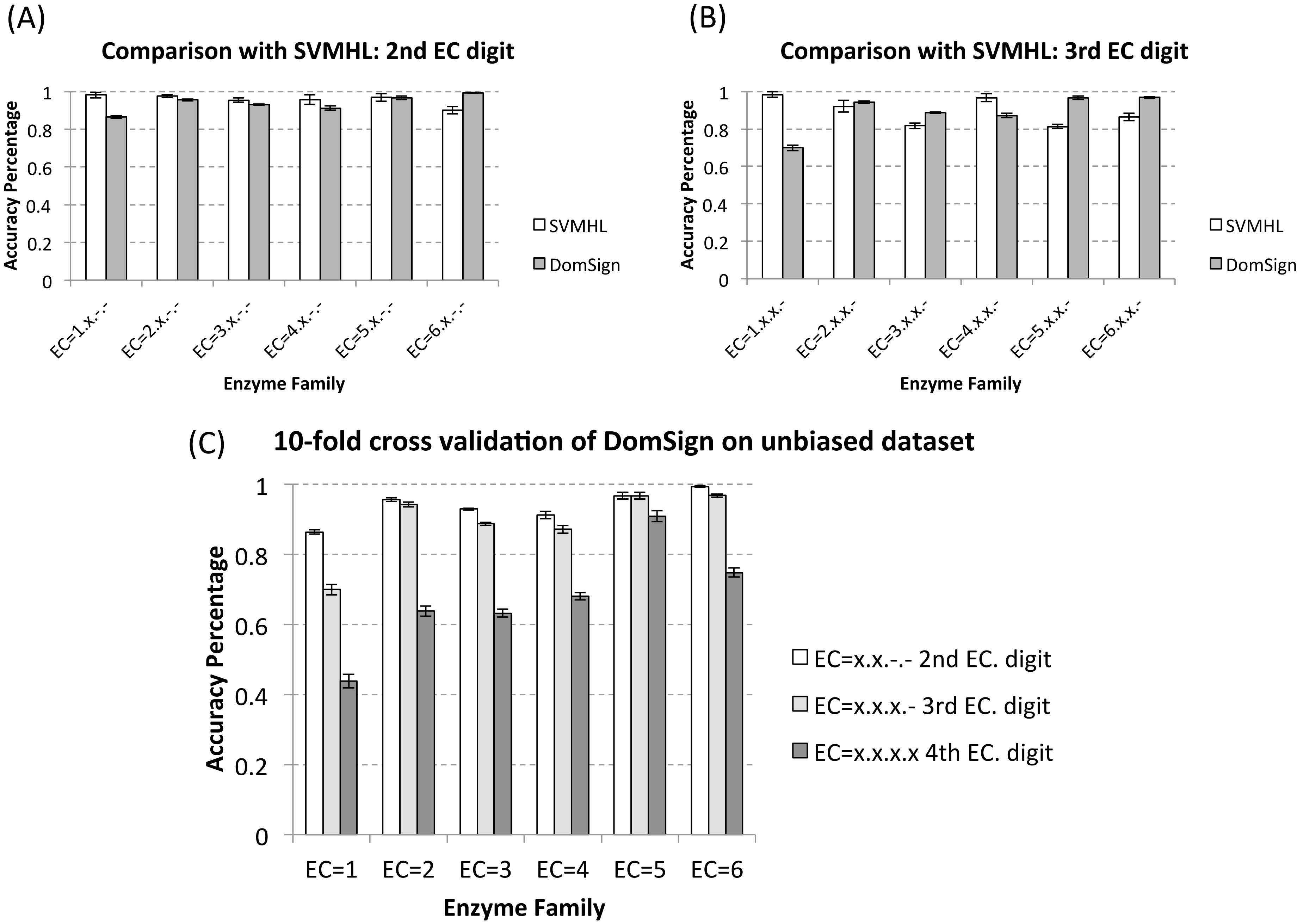

(A)
Comparison with SVMHL: 2nd EC digit
Accuracy Percentage
1
0.8
0.6
0.4
0.2
0
EC=1.x.-.-
EC=2.x.-.-
EC=3.x.-.-
EC=4.x.-.-
EC=5.x.-.-
EC=6.x.-.-
Enzyme Family
SVMHL
DomSign
(B)
Comparison with SVMHL: 3rd EC digit
Accuracy Percentage
1
0.8
0.6
0.4
0.2
0
EC=1.x.x.-
EC=2.x.x.-
EC=3.x.x.-
EC=4.x.x.-
EC=5.x.x.-
EC=6.x.x.-
Enzyme Family
SVMHL
DomSign
(C)
10-fold cross validation of DomSign on unbiased dataset
Accuracy Percentage
1
0.8
0.6
0.4
0.2
0
EC=1
EC=2
EC=3
EC=4
EC=5
EC=6
Enzyme Family
EC=x.x.-.- 2nd EC. digit
EC=x.x.x.- 3rd EC. digit
EC=x.x.x.x 4th EC. digit
