# Supplementary material for: DomSign: a top-down annotation pipeline to enlarge enzyme space in the protein universe
Source: BMC Bioinformatics. 2015 Mar 21;16:96. doi: 10.1186/s12859-015-0499-y (PMC4389672; doi:10.1186/s12859-015-0499-y)
Supplement: Additional file 12: — Enzyme prediction from HMP phase I non-redundant proteins with different specificity thresholds (99%, 90%, 80% and 70%). Stacked columns divided into different patterns refer to different EC hierarchy evels in annotation result as described in Additional file 8. The performance of DomSign with 99%, 90%, 80% and 70% specificity thresholds are compared with original HMP annotation result. HMP-PfamA-protein refers to the enzyme subset of HMP non-redundant proteins encompassing Pfam-A domains. [file 12859_2015_499_MOESM12_ESM.pdf]

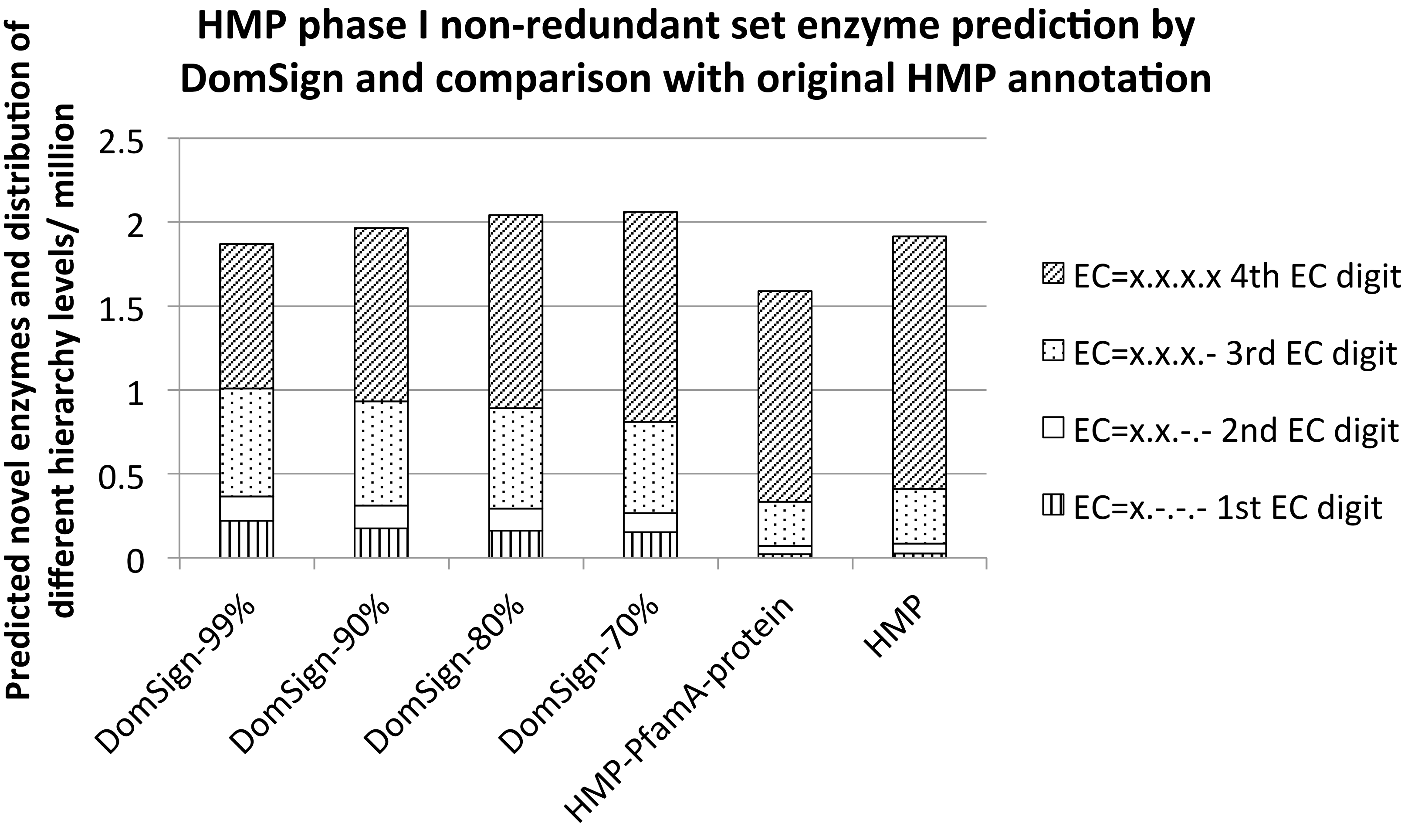
HMP phase I non-redundant set enzyme prediction by DomSign and comparison with original HMP annotation
Predicted novel enzymes and distribution of different hierarchy levels/ million
2.5
2
1.5
1
0.5
0
DomSign-99%
DomSign-90%
DomSign-80%
DomSign-70%
HMP-PfamA-protein
HMP
EC=x.x.x.x 4th EC digit
EC=x.x.x.- 3rd EC digit
EC=x.x.-.- 2nd EC digit
EC=x.-.-.- 1st EC digit
